# The accuracy of heparin-binding protein and interleukin-6 in predicting prognosis of severe pneumonia with sepsis patients

**DOI:** 10.3389/fcimb.2025.1554214

**Published:** 2025-11-10

**Authors:** Zhiyong Wei, Maimaitijiang YuSuPu, Xiaoye Wang, Yu Meng, Chunli Zhang, Xiaomeng Xue, Yan Cui, Keliang Xie

**Affiliations:** 1 Department of Critical Care Medicine, Tianjin Medical University General Hospital, Tianjin, China; 2 Department of Anesthesiology, Tianjin Institute of Anesthesiology, Tianjin Medical University General Hospital, Tianjin, China; 3 Department of Pathogen Biology, School of Basic Medical Sciences, Tianjin Medical University, Tianjin, China

**Keywords:** severe pneumonia, sepsis, heparin-binding protein, interleukin-6, C-reactive protein, procalcitonin, SOFA score

## Abstract

**Background:**

Early warning is critical for improving prognosis in patients with severe pneumonia-induced sepsis. Conventional biomarkers and the Sequential Organ Failure Assessment (SOFA) score have notable limitations in sensitivity, specificity, and timeliness. This study aimed to evaluate the prognostic value of serum heparin-binding protein (HBP) and interleukin-6 (IL-6), assess their correlation with clinical outcomes, and compare their predictive performance against traditional biomarkers and the SOFA score.

**Methods:**

A total of 171 patients with severe pneumonia complicated by sepsis were enrolled and stratified into survivor (n=96) and non-survivor (n=75) groups based on 28-day mortality. Baseline characteristics, including demographic data, comorbidities, and laboratory markers—HBP, IL-6, procalcitonin (PCT), C-reactive protein (CRP), lactate (Lac)—as well as SOFA scores, were collected upon admission. Multivariate logistic regression was performed to identify independent predictors of mortality. Predictive accuracy was assessed using receiver operating characteristic (ROC) curve analysis, with pairwise comparisons of area under the curve (AUC) conducted via DeLong’s test. Spearman’s rank correlation was used to evaluate the association between biomarker levels and organ dysfunction severity. Statistical significance was defined as P<0.05.

**Result:**

Non-survivors had significantly higher HBP, IL-6, PCT, CRP, Lac, and SOFA scores than survivors (all P<0.05). IL-6 was markedly elevated in blood culture-positive patients (P<0.05), suggesting value in detecting bloodstream infections. Multivariate analysis confirmed HBP (OR = 1.006, 95% CI:1.002–1.011), IL-6 (OR = 1.004, 95% CI:1.001–1.007), and SOFA score (OR = 1.026, 95% CI:1.145–1.484) as independent prognostic factors (all P<0.05). ROC analysis showed IL-6 had the highest AUC (0.80), followed by SOFA (0.78) and HBP (0.73), with no significant AUC differences between IL-6 and SOFA (P = 0.719) or HBP and CRP/PCT (both P>0.05). Optimal cut-offs were 55.90 ng/mL for HBP (sensitivity 82.7%, specificity 53.1%) and 32.62 pg/mL for IL-6 (sensitivity 77.3%, specificity 69.8%). HBP correlated strongest with SOFA (r=0.60, P<0.01).

**Conclusion:**

Serum HBP and IL-6 have comparable predictive efficacy to SOFA score and are numerically superior to PCT, CRP, and Lac. IL-6 also aids early identification of bloodstream infections. However, their cut-offs are from a single-center cohort and require external validation; combined use with SOFA score is recommended clinically.

## Introduction

Severe pneumonia combined with sepsis is a prevalent and highly perilous disease in clinical practice, featuring high morbidity and mortality rates, and posing a serious threat to the life and health of patients (Bewick et al., 2008). Pulmonary inflammation gives rise to a considerable number of inflammatory factors and mediators, which enter the bloodstream and trigger a series of immune responses, leading to the continuous amplification of the inflammatory response. Subsequently, it causes systemic inflammatory response syndrome, increased vascular permeability, microcirculation disorders, coagulation dysfunction and other conditions, and ultimately results in sepsis (Angus and van der Poll, 2013; Li et al., 2020). The potential circulatory, cellular and metabolic disorders in patients with severe pneumonia accompanied by sepsis can give rise to multiple organ tissue hypoperfusion, resulting in hypoxia of the cells and tissues of the body, causing organ dysfunction and significantly elevating the risk of death. Studies have demonstrated that the case fatality rate of patients with septic shock is as high as 30% - 50% (Dellinger et al., 2004; Hotchkiss et al., 2016). Consequently, early diagnosis, prognostic risk assessment and timely as well as effective intervention are of paramount importance for improving the prognosis and quality of life of patients with sepsis resulting from severe pneumonia (Font et al., 2020). At present, the clinical diagnosis and evaluation of severe pneumonia complicated with sepsis primarily depend on traditional indicators, such as PCT, CRP, and SOFA scores. Nevertheless, these traditional indicators have certain limitations in practical application, such as relatively poor specificity and sensitivity. With the intensification of medical research, numerous new biomarkers have been discovered and applied in clinical practice. Serum HBP and IL-6, as potential biomarkers, have demonstrated some value in the diagnosis and prognosis assessment of various diseases. As one of the inflammatory mediators of polymorphonuclear neutrophil leukocytes (Tapper et al., 2002), HBP is pre-synthesized and promptly released in response to early infection, it is a chemical inducer for the activation of various cells, especially monocytes (Chang et al., 2018). During infection, HBP causes leakage of the endothelium of blood vessels and modulates the inflammatory responses of numerous cell types (Lu et al., 2021). Some studies on the association between HBP and sepsis suggest that HBP may serve as an early warning sign of sepsis, particularly in patients with organ failure (Kahn et al., 2019). IL-6 is an important cytokine that is often significantly elevated in patients with pneumonia. Studies have demonstrated (Han et al., 2020) that when a person is infected with pneumonia, the body’s immune system is activated, prompting the secretion and release of numerous cytokines. Among them, IL-6 levels change conspicuously as the disease progresses. High levels of IL-6 often indicate a more complicated disease, more difficulty in treatment, and a relatively poor prognosis. Therefore, monitoring the levels of HBP and IL-6 in patients with pneumonia is of considerable clinical significance for assessing the severity of pneumonia and formulating reasonable treatment plans.

In light of this, the aim of this study was to explore the expression levels of serum HBP and IL-6, along with traditional indicators, and their diagnostic value in patients with severe pneumonia complicated with sepsis. Through comprehensive analysis of these indicators, it is anticipated to offer a more scientific and precise basis for the early diagnosis, accurate assessment of the condition and prognosis determination of severe pneumonia complicated with sepsis, thereby providing strong support for the formulation of clinical treatment decisions and further enhancing the treatment success rate and quality of life of patients.

## Methods

### Study design and patient population

The study employed a retrospective methodology. Clinical data of patients with severe pneumonia complicated with sepsis admitted to the Intensive Care Unit of Tianjin Medical University General Hospital from September 2022 to September 2024 were retrospectively analyzed. In accordance with the preset inclusion and exclusion criteria, 171 patients were selected for this study. Inclusion criteria: ① Diagnostic criteria for severe pneumonia formulated by IDSA/ATS in the United States were adopted (Lim et al., 2009).At the same time, the relevant diagnostic criteria of sepsis in “Sepsis 3.0” were met (Singer et al., 2016).② Be admitted to the intensive care unit within 24 hours after admission, and stay ≥72 hours. Exclusion criteria: ①The existence of immunodeficiency disorders (such as human immunodeficiency virus infection, blood disorders) or neutropenia (absolute neutrophil count ANC < 1.5×10^9^/L); ② Malignant tumors; ③ Concurrent with severe liver, kidney, cardiovascular and cerebrovascular diseases; ④ Death within 72 hours after admission; ⑤ Long-term utilization of antibiotics; ⑥ Concurrent with other systemic infectious diseases. The prognosis of patients within 28 days after admission was statistically based on hospitalization records or telephone or outpatient follow-up records after discharge. Survivors were those who survived from the beginning to the end of follow-up (from the start of treatment to 28 days). Those who died were those whose events (all-cause deaths) occurred from the beginning to the end of treatment. Patients who died during follow-up were included in the non-survival group by data review; survivors were included in the survival group.

### Data collection

Baseline data upon admission were collected by accessing the hospital’s electronic medical record system, encompassing gender, age, SOFA scores within 24 hours of admission, and co-existing underlying conditions (such as hypertension, diabetes, etc.). The relevant laboratory examination indicators of patients during their stay in the ICU were queried in the hospital’s electronic medical record system, and corresponding statistics were made. These comprised IL-6, HBP, SOFA scores, Acute Physiological and Chronic Health Evaluation II (APACHE II) score, CRP, Lac, PCT, and other indicators. In terms of ethics, this study adheres to the standards of medical ethics and has been approved by the Medical Ethics Committee of our Hospital (approval number:IRB2024-YX-593-01), ensuring that the rights and interests of patients and ethical principles are strictly safeguarded during the study and guaranteeing the scientificity and legitimacy of the study. This approval provides a crucial ethical basis for the smooth conduct of the study and also reflects the significance attached to the protection of patient information and ethical considerations.

### Statistical methods

In this study, SPSS 26.0 statistical software was used for comprehensive data analysis, and GraphPad Prism 9.0 was used to draw charts to visually present data characteristics. Perform normality testing for continuous variables using the Shapiro-Wilk test. The continuous variables were treated differently according to their distribution. If the continuous variable is not normally distributed, it is presented in the form of the median (quartile) [M (QL, QU)], and the Mann-Whitney U test is applied when comparing such data between two groups. Regarding the measurement data of normal distribution, the mean ± standard deviation (x ± s) is employed, and the independent sample t test is conducted. Categorical variables were presented as example (percentage), and the Chi-square test was adopted for corresponding analysis. In the multivariate analysis, factors that showed statistically significant differences in the inter-group comparative analysis were incorporated into the multivariate logistic regression analysis, in order to precisely identify the risk factors influencing the prognosis of patients with severe pneumonia complicated with sepsis. Meanwhile, the predictive efficacy of each indicator was comprehensively evaluated by drawing the ROC curve. The DeLong test was used to conduct pairwise comparisons of the area under the ROC curve (AUC) for each index to examine the differences in predictive performance among different biomarkers; all AUC values were reported with 95% confidence intervals (CI), and statistical significance was set at P < 0.05. In addition, Spearman correlation analysis was used to analyze the correlation between HBP, IL-6, PCT, CRP, Lac and SOFA scores. During the entire statistical analysis, the bilateral significance level was set at 0.05, and when *P* < 0.05, the difference was statistically remarkable.

## Results

### Baseline information

**Figure 1:**
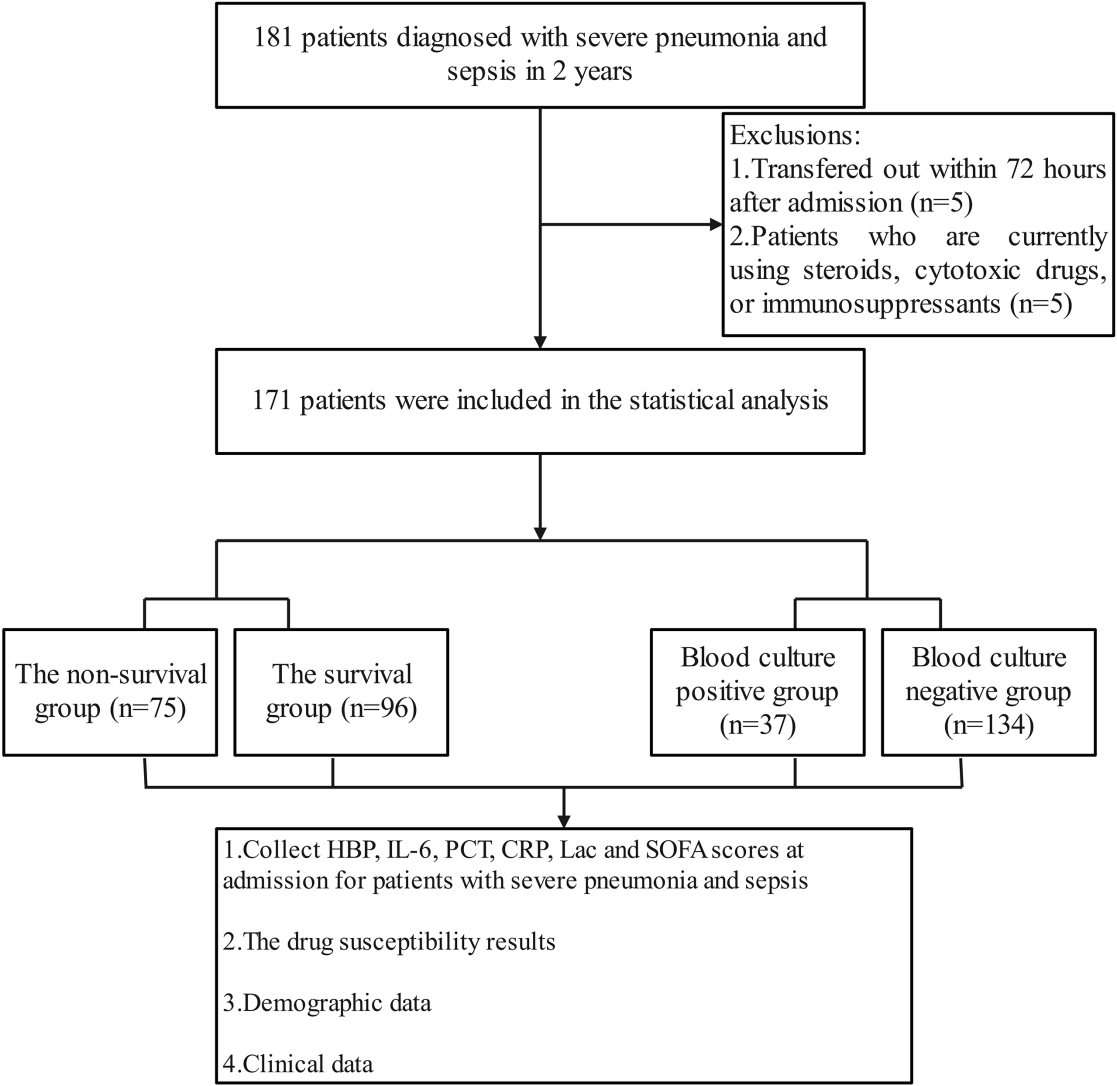
Flow chart for patient selection. A total of 181 patients with severe pneumonia and sepsis were analyzed. After excluding 10 patients, 171 patients were included for clinical data analysis.

From September 2022 to September 2024, the research team collected clinical data of 181 patients with severe pneumonia complicated by sepsis. During the data screening process, 5 patients with hospital stay shorter than 72 hours and 5 patients who had received hormone, cytotoxic drugs or immunosuppressants were excluded. Finally, 171 cases were included in the statistical analysis (**Figure 1**). Among the 171 patients with severe pneumonia complicated by septic shock included in the analysis, 112 were male (accounting for 65.5%), and 59 were female (accounting for 34.5%); the age range was 22 to 93 years, with a median age of 70 years (interquartile range of 55 to 78 years), among which 121 patients (accounting for 70.1%) were 60 years old or above, and 50 patients (accounting for 29.9%) were under 60 years old. All patients were Han Chinese residents admitted to the ICU of Tianjin Medical University General Hospital (due to the population structure characteristics of the research center location, no patients of other ethnicities were included), and there were no foreign or ethnic minority patients. During the study period, 75 cases of all-cause death occurred, and all deaths occurred during hospitalization, with a mortality rate of 43.86% (75/171). According to the 28-day prognosis, the patients were divided into the survival group (n = 96) and the death group (n = 75). Further analysis showed that there were no significant differences between the two groups in terms of age, gender, underlying diseases (including hypertension, cardiovascular diseases, kidney diseases, etc.), continuous renal replacement therapy (CRRT), deep vein catheterization, positive rate of sputum culture, etc. (P > 0.05); however, in terms of the use of bronchoscopy, the proportion of patients in the survival group who received the examination was significantly higher than that in the death group; in terms of the positive rate of blood culture, the survival group was lower than the death group; in terms of the types of pathogenic bacteria in blood culture, the proportion of Gram-negative bacteria in the survival group was lower than that in the death group; in addition, the APACHE II score of the survival group was also significantly lower than that of the death group (**Table 1**).

**Table 1:** Characteristics of the study population.

Characteristics	Survival (n=96)	Non-survival (n=75)	χ^2^/t/z value	*P*-value
Age [M(Ql,Qu)]/years	70.00(51.25,80.00)	71.00(59.00,77.00)	-0.229^c^	0.819
Male [n (%)]	62 (61.58%)	50 (66.67%)	0.081^b^	0.776
Hospital stay [d,M(Ql,Qu)]	15.00 (9.00, 26.75)	13.00 (6.00, 22.00)	-1.820^c^	0.069
Medical history [n (%)]				
Hypertension	58 (60.42%)	37 (49.33%)	2.095^b^	0.148
Diabetes mellitus	42 (43.80%)	23 (30.67%)	3.059^b^	0.080
Cardiovascular disease	25 (26.04%)	24 (32.00%)	0.731^b^	0.393
Renal disease	20 (20.83%)	17 (22.67%)	0.083^b^	0.773
Respiratory disease	4 (4.17%)	3 (4.00%)	0.003^b^	0.956
Malignant tumor	16 (16.67%)	17 (22.67%)	0.973^b^	0.324
CRRT [n (%)]	17 (17.71%)	17 (22.67%)	0.650^b^	0.420
Deep vein catheterization [n (%)]	91 (94.79%)	74 (98.67%)	1.867^b^	0.172
Bronchofibroscope [n (%)]	63 (65.63%)	31 (41.33%)	5.213^b^	0.022*
Sputum culture positive [n (%)]	86 (89.58%)	64 (85.33%)	0.706^b^	0.401
Blood culture positive [n (%)]	12 (12.50%)	25 (33.33%)	10.778^b^	0.001*
Blood culture positive pathogen [n (%)]				
Gram-negative bacterium	5 (5.21%)	16 (21.33%)	10.163^a^	0.001*
Gram-positive bacterium	7 (7.29%)	8 (10.67%)	0.599^a^	0.439
Fungus	2 (2.08%)	2 (2.67%)	0.063^a^	0.802
APACHE II score [M(Ql,Qu)]	19.00 (15.00, 24.00)	23.00 (18.00, 31.00)	-3.119^c^	0.002*
Septic shock (n%)	31 (32.29%)	37 (49.33%)	5.105	0.024*

CRRT, continuous renal replacement therapy; n, numbers; APACHE II, Acute Physiology and Chronic Health Evaluation II; SEM, mean ± standard error of mean; t: Student t-test; χ2, Chi-square test; z: Rank-sum test. *P*-value: <0.05 was considered as statistically significant.a for t, b for χ2 ;c for z.*Values that with significant differences.

### Isolated organisms

In this study, 39 patients presented with positive blood cultures (**Table 2**). Overall, gram-negative bacteria constituted the main cause of most infections. In the blood culture, a diverse range of microorganisms were detected, including *Staphylococcus epidermidis* 6 times, *Staphylococcus hominis* 6 times. *Klebsiella pneumoniae* occurred more frequently, reaching up to 13 times; furthermore, *Acinetobacter baumannii* appeared 4 times, and *Candida albicans* occurred 3 times. The presence of these microorganisms in the blood could indicate different infection statuses and potential risks. Sputum culture differed. *Klebsiella pneumoniae* occurred 41 times, *Candida albicans* 37 times, and *Acinetobacter baumannii* 32 times. *Pseudomonas aeruginosa* occurred 19 times, etc.

**Table 2:** Organisms isolated from blood and sputum.

Isolated organisms	Blood cultures	Sputum cultures
*Candida albicans*	3	37
*Klebsiella pneumoniae*	13	41
*Stenotrophomonas maltophilia*	2	13
*Pseudomonas aeruginosa*	3	19
*Acinetobacter baumannii*	4	32
*Staphylococcus aureus*	0	5
*Aspergillus fumigatus*	0	5
*Serratia marcescens*	0	2
*Corynebacterium*	0	4
*Staphylococcus epidermidis*	6	0
*Escherichia coli*	1	0
*Enterobacter hormeri*	1	0
*Candida parapsilosis*	1	0
*Staphylococcus hominis*	6	0
*Hemolytic staphylococcus*	1	0
*Clostridium perfringens*	2	0
*Coccus glucosus*	1	0

### Comparison of laboratory examination indexes and organ function impairment scores between survival group and non-survival group

**Figure 2:**
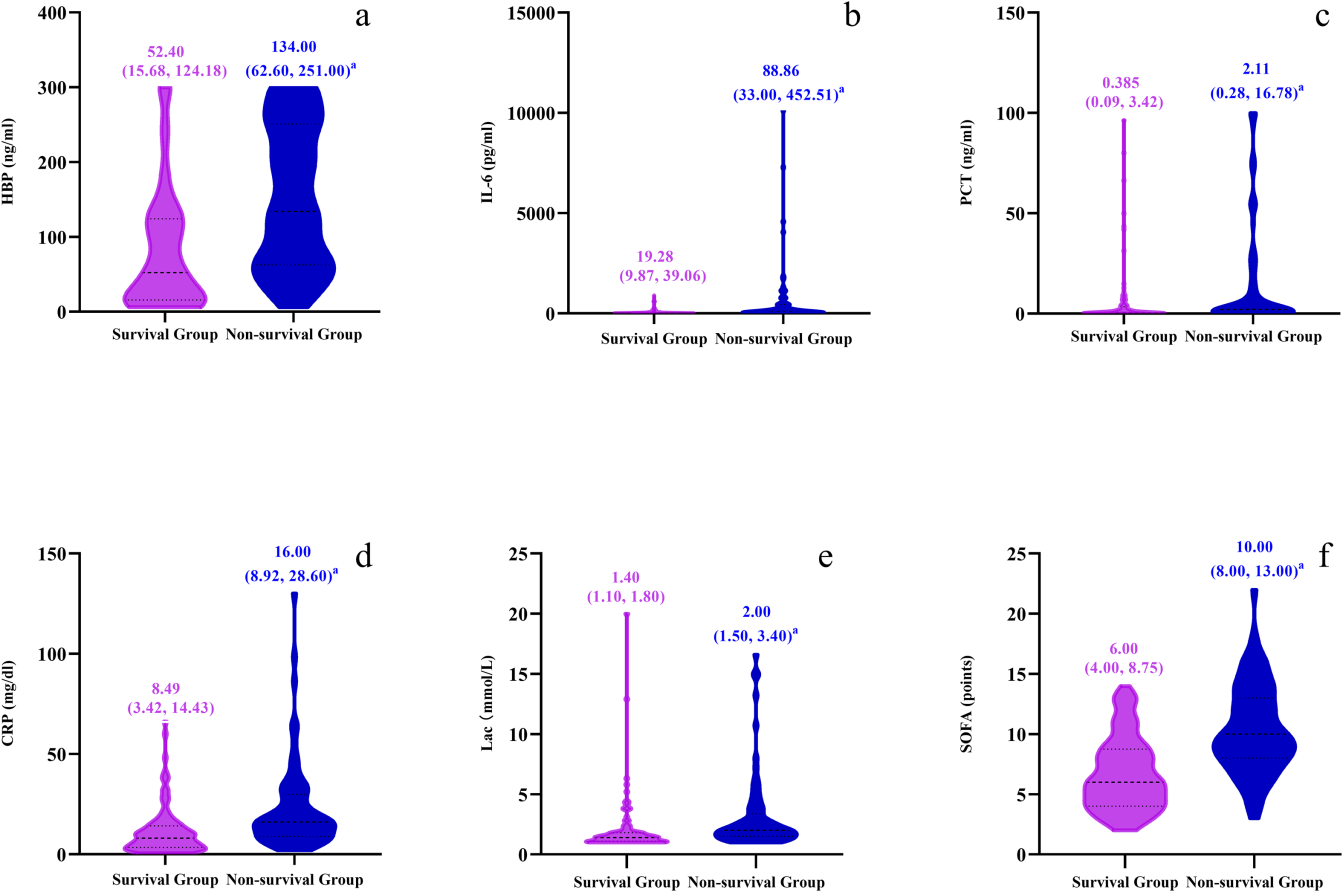
Violin plots compare survival and non-survival groups across six parameters: **(a)** HBP (ng/ml), **(b)** IL-6 (pg/ml), **(c)** PCT (ng/ml), **(d)** CRP (mg/dl), **(e)** Lac (mmol/L), and **(f)** SOFA points. Plots display median values and interquartile ranges.

**Figure 3:**
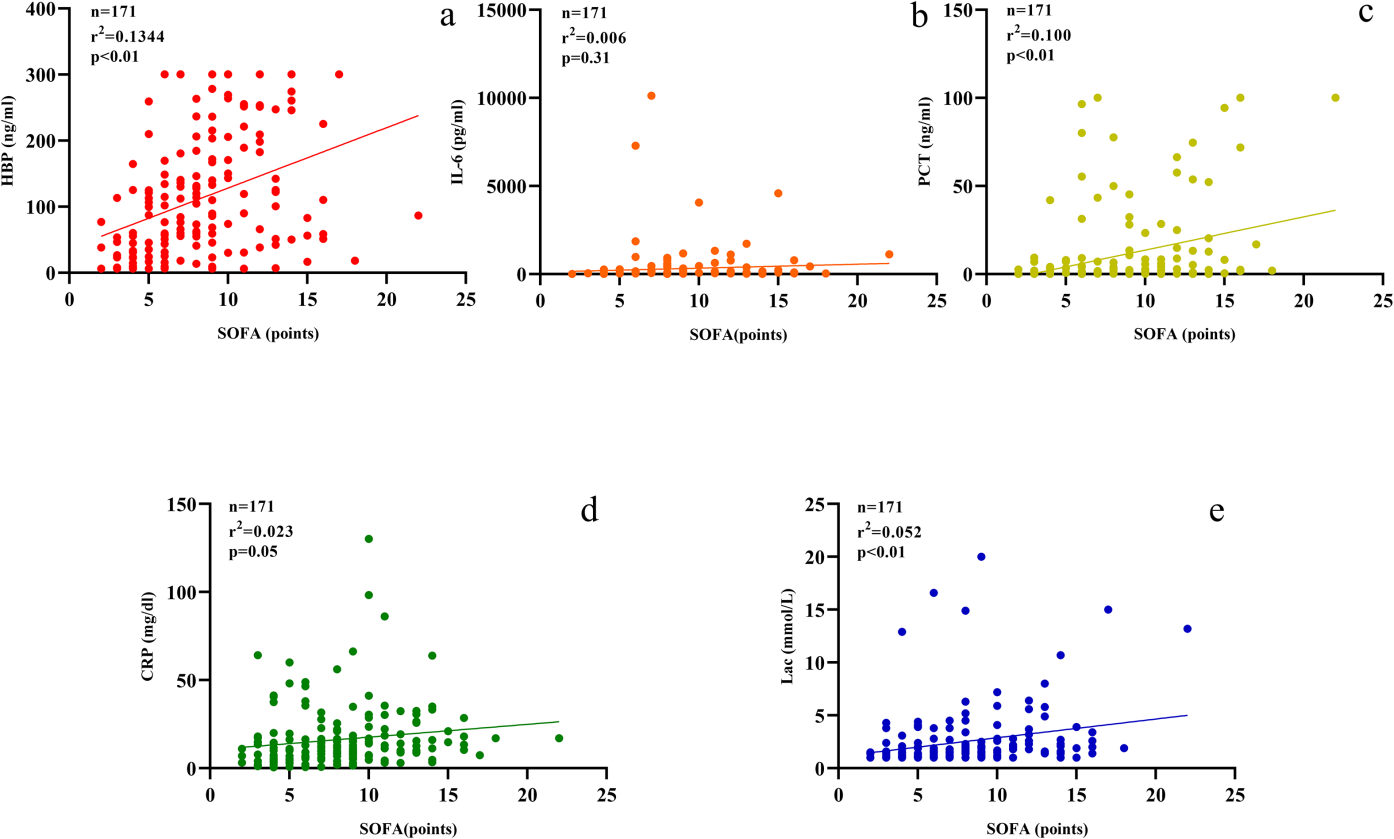
Scatter plots **(a–e)** display relationships between SOFA scores and various biomarkers. **(a)** HBP; **(b)** IL-6; **(c)** PCT; **(d)** CRP; **(e)** Lactate. Each plot includes sample size, r-squared, and p-values.

**Figure 4:**
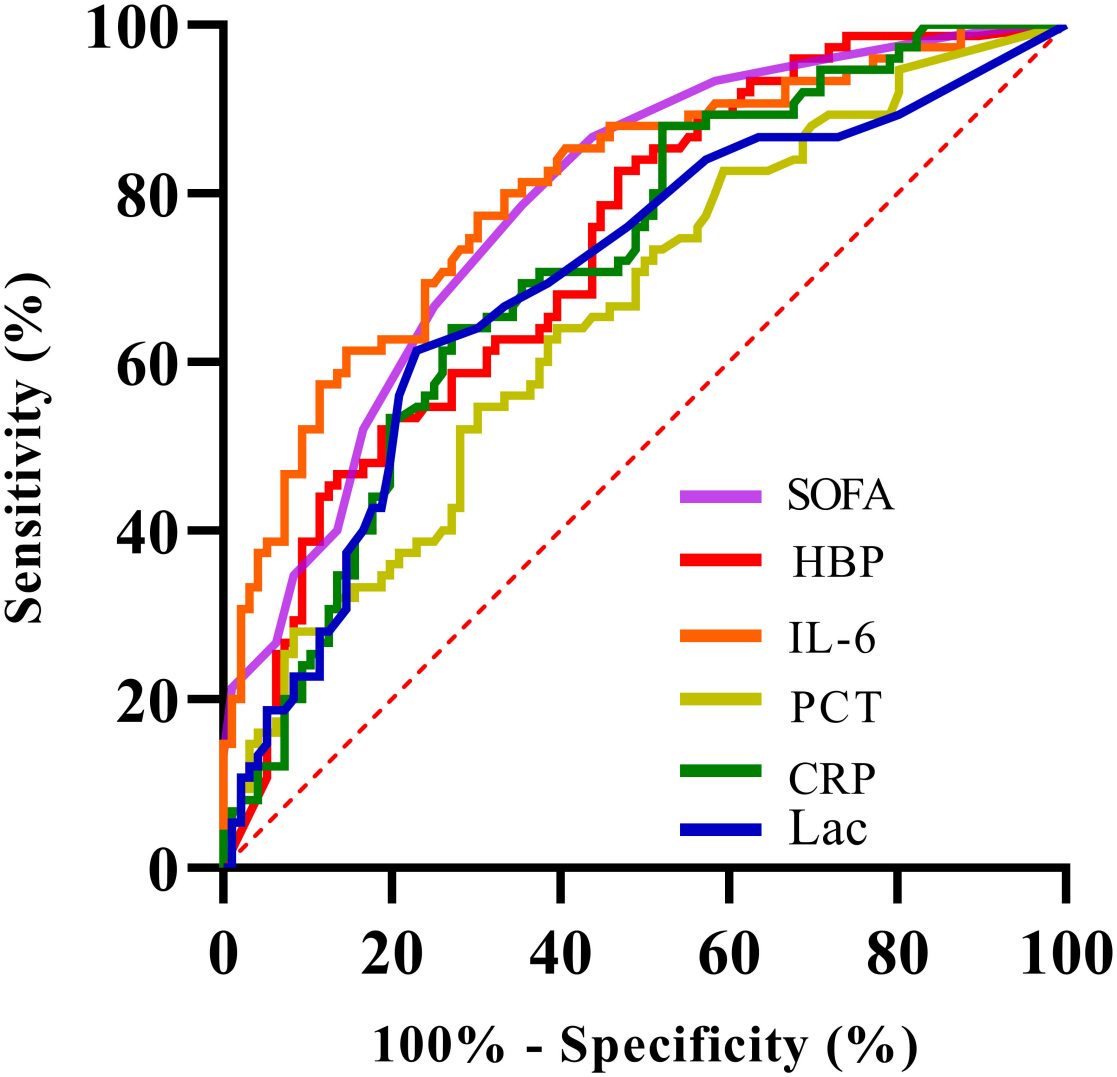
Receiver Operating Characteristic (ROC) curve showing sensitivity versus 100% minus specificity for various biomarkers: SOFA (purple), HBP (red), IL-6 (orange), PCT (yellow), CRP (green), and Lac (blue). The diagonal red dashed line represents the line of no discrimination.

The levels of HBP, IL-6, Lac, PCT, CRP and SOFA scores in the non-survival group were conspicuously higher than those in the survival group (all *P* < 0.05) (**Figure 2**). A logistic regression analysis was conducted on the risk factors influencing the prognosis of patients with severe pneumonia complicated with sepsis (**Table 3**). Logistic regression analysis disclosed that HBP ( OR = 1.006, 95% CI:1.002 ~ 1.011, P < 0.05), IL-6 ( OR = 1.004, 95% CI:1.001 ~ 1.007, P < 0.05 ) and SOFA ( OR = 1.026, 95% CI: 1.145 ~ 1.484, P < 0.05 ) scores were independent risk factors affecting sepsis (all P < 0.05), for every one-unit increase in HBP, IL-6 levels, and SOFA score, the risk of 28-day mortality in patients increases by 0.6%, 0.4%, and 2.6%, respectively. Correlation analysis regarding HBP, IL-6, PCT, CRP, Lac and SOFA score (**Table 4**; **Figure 3**): Spearman correlation analysis manifested that HBP, PCT and Lac were significantly and positively correlated with SOFA score (r^2^ values were 0.1344,0.100,0.052 respectively, all *P* < 0.01), among which HBP had the most robust correlation. The prognostic value of HBP, IL-6, PCT, CRP, Lac and SOFA scores in patients with severe pneumonia complicated with sepsis (**Table 5**; **Figure 4**): ROC curve analysis showed that IL-6 had the greatest area under the receiver operating characteristic curve, which was 0.80 (95% confidence interval 0.71 - 0.87), HBP was 0.73 (95% confidence interval 0.66 - 0.81), and SOFA score was 0.78 (95% confidence interval 0.72 - 0.85). Lac was 0.70 (95% CI 0.62 to 0.78), PCT was 0.66 (95% CI 0.57 to 0.74), and CRP was 0.72 (95% CI 0.64 to 0.79). When the cutoff level of HBP was set at 55.9 ng/mL, the sensitivity reached 82.7% and the specificity was 53.1%. When the cutoff level of IL-6 was 32.62 pg/mL, the sensitivity stood at 77.3% and the specificity was 69.8%.

**Table 3:** Multivariate logistic analysis of the risk factors predicting 28-day death in patients with severe pneumonia and sepsis.

Variable	β	S.E.	Wald χ²	OR value	95%CI	P-value
HBP	0.006	0.002	7.537	1.006	1.002~1.011	<0.05
IL-6	0.004	0.002	6.276	1.004	1.001~1.007	<0.05
PCT	0.001	0.010	0.005	1.001	0.981~1.021	0.945
Lac	0.026	0.087	0.087	1.026	0.866~1.216	0.768
CRP	0.024	0.014	2.827	1.024	0.996~1.054	0.093
SOFA score	0.265	0.066	16.045	1.026	1.145~1.484	<0.05

HBP, heparin-binding protein; IL-6, Interleukin-6; PCT, procalcitonin; Lac, Lactic acid; CRP, C‐reactive protein; SOFA, Sequential Organ Failure Assessment score.

**Table 4:** Correlation analysis between HBP, IL-6, PCT, CRP, Lac and SOFA score.

Indicator	SOFA score		
	r^2^ value	95%CI	*P *value
HBP	0.1344	5.615~12.66	<0.01
IL-6	0.006	-20.99~65.96	0.31
PCT	0.1	1.029~2.753	<0.01
CRP	0.023	0.0005~1.454	0.05
Lac	0.052	0.06264~0.29	<0.01

SOFA, Sequential Organ Failure Assessment score; HBP, heparin-binding protein; IL-6, Interleukin-6; PCT, procalcitonin; CRP, C‐reactive protein; Lac, Lactic acid.

**Table 5:** The prognostic significance of HBP, IL-6, PCT, CRP, Lac, and SOFA for patients diagnosed with severe pneumonia and sepsis.

Indicator	Critical value	Sensitivity%	Specificity%	AUC	95%CI	*P* value
HBP	55.90	82.7	53.1	0.73	0.66~0.81	<0.01
IL-6	32.62	77.3	69.8	0.80	0.71-0.87	<0.01
PCT	1.825	54.67	69.79	0.66	0.57-0.74	<0.01
CRP	13.5	64	72.92	0.72	0.64-0.79	<0.01
Lac	1.85	61.33	77.08	0.70	0.62-0.78	<0.01
SOFA	7.5	78.67	64.58	0.78	0.72-0.85	<0.01

SOFA, Sequential Organ Failure Assessment score; HBP, heparin-binding protein; IL-6, Interleukin-6; PCT, procalcitonin; CRP, C‐reactive protein; Lac, Lactic acid.

The Delong test was used to conduct pairwise comparisons of the AUC values for each indicator: 1. The AUC of IL-6 was significantly higher than that of PCT (Z = 3.277, P = 0.001 < 0.05), Lac (Z = 2.169, P = 0.03 < 0.05), but there was no significant difference compared with CRP (Z = 1.867, P = 0.062 > 0.05), HBP (Z = 1.367, P = 0.172 > 0.05), and SOFA score (Z = 0.360, P = 0.719 > 0.05). 2. The AUC of HBP was not significantly different from CRP (Z = 0.316, P = 0.752 > 0.05), Lac (Z = 0.615, P = 0.515 > 0.05), IL-6 (Z = -1.367, P = 0.172 > 0.05), PCT (Z = 1.358, P = 0.174 > 0.05), and SOFA score (Z = -1.209, P = 0.227 > 0.05). There was no significant difference in the AUC comparisons among CRP, Lac, and PCT (all P > 0.05).

### Value of serum HBP and IL-6 in the identification of patients with positive blood cultures

**Figure 5:**
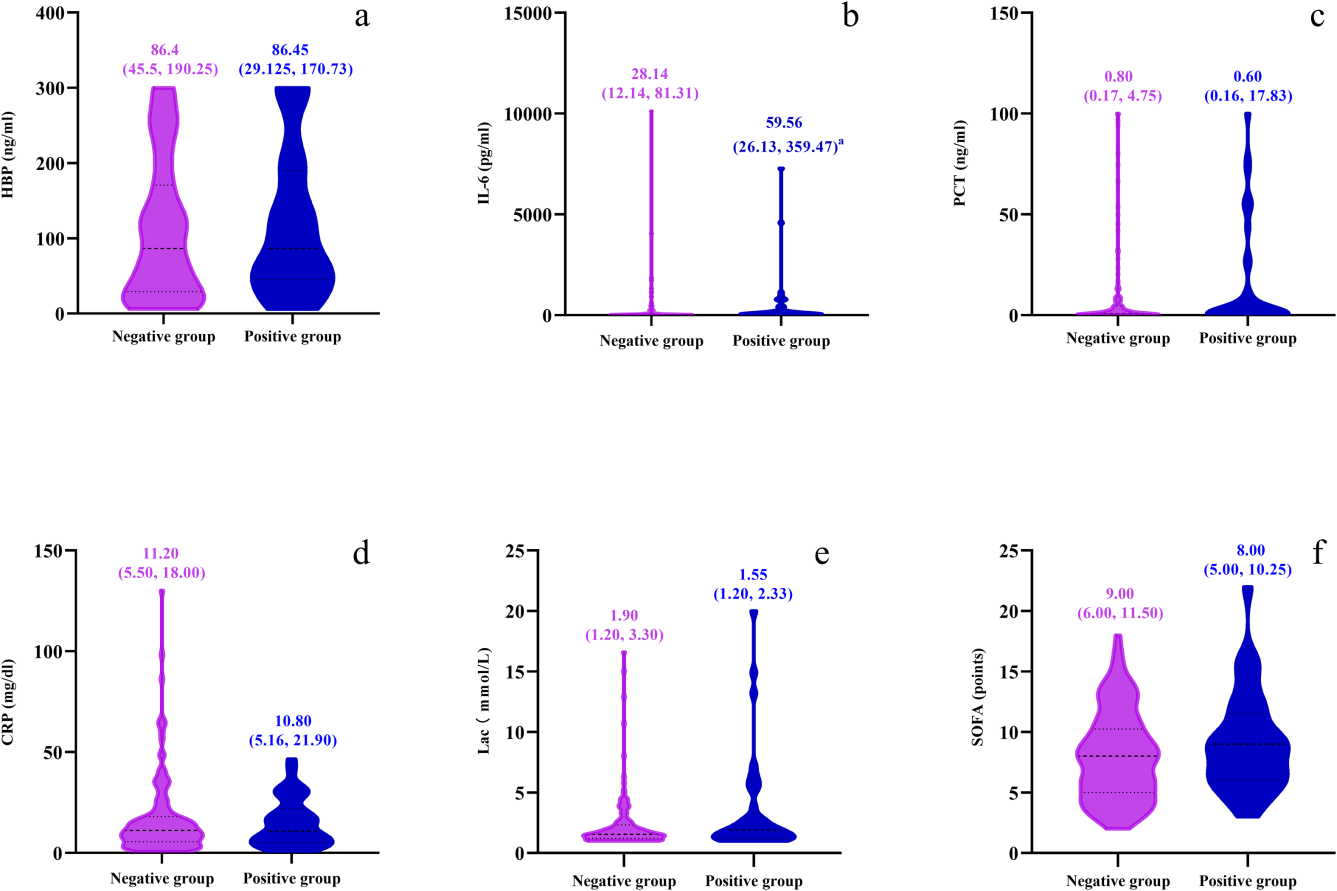
Violin plots depicting comparisons between negative and positive groups for six parameters: **(a)** HBP (ng/ml), **(b)** IL-6 (pg/ml), **(c)** PCT (ng/ml), **(d)** CRP (mg/dl), **(e)** Lactate (mmol/L), and **(f)** SOFA points. Each plot displays median values with interquartile ranges for both groups using distinct colors for clarity.

Based on the detection and analysis of bacteria in the blood, this study made an in-depth comparison between the patient population with positive blood culture (n=37) and that with negative blood culture (n=134). Among the patients with positive blood culture, only the index of IL-6 was significantly higher than that of the patients with negative blood culture, and the difference between the two was highly significant and had strong statistical significance (*P* < 0.01). However, when comparing serum HBP, PCT, CRP, Lac and SOFA score between the patients with positive blood culture and those with negative blood culture, none showed statistical significance (*P* > 0.05) (**Figure 5**).

## Discussion

Severe pneumonia complicated with sepsis is a challenging issue in the field of clinical critical care medicine. Characterized by high morbidity and mortality rates (30%-50%) (Hotchkiss et al., 2016), it makes “early identification of high-risk patients and accurate prognosis assessment” a core requirement for improving clinical outcomes. Currently, traditional biomarkers such as procalcitonin (PCT) and C-reactive protein (CRP) relied on in clinical practice have limitations including insufficient specificity and high susceptibility to non-infectious factors (Pierrakos et al., 2020), failing to meet the needs of early intervention for critically ill patients. Although the Sequential Organ Failure Assessment (SOFA) score is the gold standard for evaluating organ failure, it requires the integration of multi-system indicators, is time-consuming, and lacks convenience for dynamic monitoring (Seymour et al., 2016). Against this backdrop, this study focused on serum heparin-binding protein (HBP) and interleukin-6 (IL-6), and systematically explored their value in prognostic assessment of patients with severe pneumonia complicated with sepsis by comparing them with traditional indicators, aiming to provide a more efficient biomarker option for clinical practice.

Through a retrospective analysis of 171 patients, this study first identified significant differences in prognostic indicators between groups: levels of HBP, IL-6, lactate (Lac), PCT, CRP, and SOFA scores in the non-survival group were significantly higher than those in the survival group (all P < 0.05), with a higher positive rate of blood culture and a higher proportion of gram-negative bacterial infections. These findings suggest that these indicators are directly associated with disease severity and poor outcomes. Further multivariate logistic regression analysis confirmed that HBP (OR = 1.006, 95% CI: 1.002-1.011), IL-6 (OR = 1.004, 95% CI: 1.001-1.007), and SOFA score (OR = 1.026, 95% CI: 1.145-1.484) were independent risk factors affecting the 28-day mortality risk of patients. For each unit increase in these three indicators, the mortality risk increased by 0.6%, 0.4%, and 2.6% respectively. This result directly established the core position of HBP and IL-6 in prognostic assessment. Correlation analysis further revealed the association between indicators and organ damage: HBP, PCT, and Lac were all positively correlated with SOFA scores (r² = 0.1344,0.100,0.052 respectively, all P < 0.01), among which HBP showed the strongest correlation, indicating its closest association with the degree of organ dysfunction. This finding is highly consistent with the pathological mechanism of HBP “directly damaging the vascular endothelial barrier” (Bentzer et al., 2016). To clarify the differences in predictive efficacy among various indicators, this study conducted pairwise comparisons of the area under the receiver operating characteristic curve (AUC) using the Delong test, and finally divided the indicators into three tiers: the optimal tier included IL-6 (AUC = 0.80, 95% CI: 0.71-0.87) and SOFA score (AUC = 0.78, 95% CI: 0.72-0.85), which were significantly superior to the third tier including PCT (AUC = 0.66, 95% CI: 0.57-0.74) and Lac (AUC = 0.70, 95% CI: 0.62-0.78); the intermediate tier included HBP (AUC = 0.73, 95% CI: 0.66-0.81) and CRP (AUC = 0.72, 95% CI: 0.64-0.79), which showed no statistical difference from the other tiers but demonstrated potential superior to traditional indicators in terms of numerical values. This tiered result verified the advantages of HBP and IL-6 from a statistical perspective, providing a clear basis for clinical indicator selection.

From the perspective of pathophysiological mechanisms, HBP and IL-6 are not merely “inflammatory accompanying indicators” but key mediators directly involved in the core pathological process of sepsis, which is the core reason for their accurate association with prognosis. HBP initially attracted attention due to its antibacterial properties (Shafer et al., 1984), but subsequent studies confirmed that its core role in sepsis lies in “damaging the vascular endothelial barrier”: Linder et al (Linder et al., 2015). found that HBP can induce endothelial cells to express intercellular adhesion molecule-1 (Ruan et al., 2002), promoting calcium ion influx to trigger endothelial cytoskeleton rearrangement and intercellular gap formation, ultimately increasing vascular permeability (Bentzer et al., 2016). The disruption of endothelial barrier integrity is the initiating link of microcirculatory disorders and multiple organ damage in sepsis (Opal and van der Poll, 2015). The strongest correlation between HBP and SOFA scores in this study essentially reflects its mechanism of “directly associating with vascular damage”: high levels of HBP exacerbate vascular leakage, leading to the infiltration of inflammatory cells and plasma components into the tissue space, causing tissue edema and organ dysfunction. This also explains why patients with high HBP levels at admission are more likely to develop multiple organ dysfunction syndrome (MODS) and have a longer length of stay in the intensive care unit (ICU) (Fisher and Linder, 2017; Yang et al., 2019). More importantly, the “ultra-early elevation” characteristic of HBP (significant increase within 6 hours of infection) makes it an ideal indicator for “ultra-early admission screening”: compared with PCT, which takes 12–24 hours to reach its peak (Yang et al., 2019), and Lac, which only reflects late hypoxia, HBP can identify sepsis risks in advance when traditional indicators have not yet changed. For example, for patients with severe pneumonia admitted to the emergency department, if HBP > 55.9 ng/mL (the cut-off value in this study), even if PCT and CRP are normal, it is necessary to initiate broad-spectrum antibiotic therapy and fluid resuscitation. This application value is also supported by Holub and Beran (Holub and Beran, 2012), who confirmed that the early identification sensitivity of HBP in patients with severe sepsis is significantly higher than that of traditional indicators.

As a multifunctional cytokine, IL-6 is secreted by a variety of cells such as macrophages and T cells (Tanaka et al., 2014), and promotes the progression of severe pneumonia complicated with sepsis through three mechanisms: first, inducing the release of inflammatory mediators such as tumor necrosis factor-α and interleukin-1β to amplify the inflammatory cascade; second, promoting the differentiation of T cells into Th17 cells to regulate the balance of immune response; third, promoting the production of coagulation factors to cause coagulation dysfunction (McElvaney et al., 2021). This characteristic of “participating in the pathological process through multiple links” enables IL-6 levels to reflect the severity of the disease in real time. In this study, IL-6 had the highest AUC (0.80) and showed no significant difference from the SOFA score (Z = 0.360, P = 0.719), indicating that it can replace the “SOFA score requiring multi-system evaluation” through convenient serum testing, providing an efficient tool for rapid risk stratification in the ICU (e.g., determining mortality risk within 1 hour of admission). In addition, the unique value of IL-6 is also reflected in “treatment response monitoring”: Song et al (Song et al., 2019). confirmed that when the level of IL-6 decreases by more than 50% after treatment in patients with severe pneumonia complicated with sepsis, the 28-day survival rate can reach 78.2%, while it is only 31.5% in patients with persistently elevated IL-6. This study further determined the clinical cut-off value of IL-6 as 32.62 pg/mL (sensitivity 77.3%, specificity 69.8%). This threshold can not only effectively identify high-risk patients but also serve as a quantitative basis for adjusting treatment regimens. If IL-6 continues to increase or decreases by < 30% during treatment, it indicates poor therapeutic efficacy, requiring re-evaluation of the infection source (e.g., drug-resistant bacteria) or enhanced organ support (e.g., optimizing mechanical ventilation, initiating continuous renal replacement therapy (CRRT)); if it decreases by > 50% within one week, the intensity of treatment can be considered to be reduced to avoid over-intervention.

The results of this study further highlight the shortcomings of traditional indicators such as PCT, CRP, and Lac: their AUC values are only 0.66-0.72, with no significant differences in pairwise comparisons, failing to meet the “early and accurate” assessment requirements. Specifically, PCT is susceptible to interference from non-infectious stress (such as surgery and trauma) or atypical pathogen infections (Schuetz et al., 2018). Christ-Crain et al (Christ-Crain et al., 2006). found that its false positive rate can reach 23%-35% in patients with community-acquired pneumonia; CRP cannot distinguish between “infectious inflammation and autoimmune inflammation”, often leading to confusion in clinical practice such as “elevated CRP without evidence of infection”; Lac only reflects late tissue hypoxia and cannot indicate risks in the early stage of the disease. Opal et al (Opal et al., 2013)once pointed out that the limitations of traditional biomarkers have become a bottleneck in the precise diagnosis and treatment of sepsis. However, HBP and IL-6 in this study exactly fill this gap: the “ultra-early warning” feature of HBP and the “inflammation quantification” feature of IL-6 solve the problems of “delayed response” and “insufficient specificity” of traditional indicators respectively, forming a complementary assessment system.

This study also observed a key phenomenon: among patients with positive blood culture, only the level of IL-6 was significantly higher than that in the negative group (P < 0.01), while there were no statistical differences in HBP, PCT, CRP, Lac, and SOFA scores. This result suggests the unique value of IL-6 in “early identification of bloodstream infections”. A study by Gille et al (Gille et al., 2021). also confirmed that the elevation of IL-6 in patients with positive blood culture is directly associated with a “more intense systemic inflammatory response”. However, other indicators may fail to sensitively capture bloodstream infection signals due to “different infection stages” (e.g., when local infection progresses to bloodstream infection, HBP only reflects local vascular damage) or “differences in pathogen types” (e.g., PCT does not increase significantly in gram-positive bacterial infections). Therefore, in clinical practice, monitoring IL-6 can detect risks earlier in patients with severe pneumonia suspected of having bloodstream infections; if combined with HBP (for evaluating microcirculation) and SOFA score (for evaluating organ damage), a more comprehensive understanding of the patient’s infection status and immune status can be obtained, avoiding the limitations of a single indicator.

Based on the results of this study, it is recommended to apply HBP and IL-6 in clinical practice according to the following scenarios to maximize their assessment value: for patients with severe pneumonia admitted to the emergency department or hospital, HBP should be tested first. If HBP > 55.9 ng/mL (sensitivity 82.7%), regardless of whether traditional indicators are abnormal, the patient should be included in the management of high-risk sepsis populations, and SOFA scores should be dynamically monitored within 24 hours to avoid early missed diagnosis; IL-6 should be tested at least twice a week, with 32.62 pg/mL as the threshold, and therapeutic efficacy should be judged based on the degree of decrease: a continuous increase or a decrease of < 30% indicates poor efficacy, requiring adjustment of antibiotics or enhanced organ support; a decrease of > 50% indicates improved condition, and consideration can be given to reducing the intensity of treatment; patients can be divided into high-risk (two or more positive indicators), medium-risk (one positive indicator), and low-risk (all indicators negative) based on IL-6 (> 32.62 pg/mL), HBP (> 55.9 ng/mL), and SOFA score (≥ 8 points): high-risk patients should be given priority in ICU resource allocation (vital signs monitored every 4 hours, organ function evaluated daily), medium-risk patients should be closely followed up for blood culture results, and low-risk patients can have the monitoring interval appropriately extended to improve the efficiency of medical resource utilization.

While this study confirms the clinical value of serum heparin-binding protein (HBP) and interleukin-6 (IL-6) in patients with severe pneumonia complicated by sepsis, several limitations should be acknowledged: First, the retrospective design inevitably leads to incomplete data collection and selection bias, which may restrict the generalizability of our findings. Second, the relatively small sample size (171 cases) undermines the stability of subgroup analyses (e.g., biomarker differences across patients with different pathogenic infections). Third, our analysis was limited to HBP, IL-6, and a few conventional markers, excluding other key inflammatory mediators such as tumor necrosis factor-α and interleukin-8. Additionally, the lack of serial monitoring data prevents in-depth exploration of associations between dynamic changes in these biomarkers and prognosis—for example, whether early reductions in HBP reflect effective neutrophil regulation. Notably, the predictive cutoffs identified for HBP (55.90 ng/mL) and IL-6 (32.62 pg/mL) are derived solely from a single-center retrospective cohort of Han Chinese patients at Tianjin Medical University General Hospital (median age 70 years, 65.5% male). These thresholds may be influenced by center-specific factors, including local clinical practices, detection platforms, and potential recruitment biases. Variations in detection methods (e.g., assay kits, instruments) across centers could also cause systematic differences in measured concentrations, leaving the generalizability of these cutoffs to other populations or laboratories unvalidated. To address these limitations, future research should focus on five areas: (1) Conduct prospective studies with standardized inclusion criteria and data collection protocols to reduce bias; (2) Expand sample sizes through multicenter collaborations, incorporating diverse populations (e.g., elderly patients, immunocompromised individuals) to enhance representativeness; (3) Investigate the mechanisms of HBP and IL-6 (e.g., interactions with neutrophils and endothelial cells) to inform therapeutic targets such as IL-6 receptor antagonists; (4) Develop dynamic prediction models integrating multi-dimensional indices (e.g., oxygenation index, duration of mechanical ventilation) to facilitate precision stratification and personalized treatment; (5) Prioritize multicenter validation of HBP and IL-6 cutoffs to promote their standardization and clinical application.

## Conclusion

This study focused on patients with severe pneumonia-induced sepsis to investigate the roles of serum HBP and IL-6, along with traditional indicators. The results indicated that higher levels of both HBP and IL-6, along with a higher SOFA score, were independent prognostic risk factors for non-survivors. In terms of prognosis prediction, IL-6 and HBP performed better than PCT, CRP, and Lac by achieving a considerable balance of sensitivity and specificity at the established cutoffs. This enables early identification of patients with poor prognoses for timely treatment adjustments. Among patients with positive blood cultures, only IL-6 showed significant elevation, highlighting its value in early infection recognition. Although other indicators did not show significant differences between groups, they are beneficial when used in combination to assess overall patient status. However, limitations such as a retrospective design and small sample size exist. Future research should be prospective and multicenter in order to delve deeper into biological mechanisms and construct multi-factor prediction models for refined diagnosis and prognosis assessment to enhance treatment efficacy and improve patient outcomes.

## Data Availability

The original contributions presented in the study are included in the article/supplementary material. Further inquiries can be directed to the corresponding authors.
